# A Potential Role of Keratinocyte-Derived Bilirubin in Human Skin Yellowness and Its Amelioration by Sucrose Laurate/Dilaurate

**DOI:** 10.3390/ijms23115884

**Published:** 2022-05-24

**Authors:** Bin Fang, Patrick D. Card, Junjun Chen, Lijuan Li, Timothy Laughlin, Bradley Jarrold, Wenzhu Zhao, Adam M. Benham, Arto T. Määttä, Timothy J. Hawkins, Tomohiro Hakozaki

**Affiliations:** 1 The Procter & Gamble Company, Mason Business Center, Mason, OH 45040, USA; fang.b@pg.com (B.F.); chen.j.96@pg.com (J.C.); li.l.28@pg.com (L.L.); laughlin.lt@pg.com (T.L.); jarrold.bb@pg.com (B.J.); zhao.wh@pg.com (W.Z.); 2 Department of Biosciences, Durham University, Stockton Road, Durham DH1 3LE, UK; patrick.d.card@durham.ac.uk (P.D.C.); adam.benham@durham.ac.uk (A.M.B.); arto.maatta@durham.ac.uk (A.T.M.); t.j.hawkins@durham.ac.uk (T.J.H.)

**Keywords:** bilirubin, keratinocyte, oxidative stress, skin yellowness, skin tone, skin color, sucrose laurate, sucrose dilaurate

## Abstract

Sallow and/or dull skin appearance is greatly attributable to the yellow components of skin tone. Bilirubin is a yellow chromophore known to be made in the liver and/or spleen and is transported throughout the body via the blood stream. Recent publications suggest bilirubin may be synthesized in other cells/organs, including the skin. We found human keratinocytes express the transcripts involved in bilirubin biosynthesis. In parallel, we also found human keratinocytes could indeed synthesize bilirubin in monolayer keratinocytes and in a 3D human skin-equivalent model. The synthesized amount was substantial enough to contribute to skin yellowness. In addition, oxidative stress enhanced bilirubin production. Using UnaG, a protein that forms a fluorescent species upon binding to bilirubin, we also visualized the intracellular expression of bilirubin in keratinocytes. Finally, we screened a compound library and discovered that the sucrose laurate/dilaurate (SDL) combination significantly reduced bilirubin levels, as well as bilirubin-mediated yellowness. In conclusion, bilirubin is indeed synthesized in epidermal keratinocytes and can be upregulated by oxidative stress, which could contribute to chronic or transient yellow skin tone appearance. Application of SDL diminishes bilirubin generation and may be a potential solution to mitigate yellowish and/or dull skin appearance.

## 1. Introduction

Yellow skin color is frequently associated with sallow and/or dull skin appearance, which is considered a barometer of poor health. Coincidentally, dull skin appearance can be exacerbated by conditions that commonly lead to increased inflammation and oxidative stress, such as sleep deprivation, acute and/or chronic mental or physical stresses, and poor diet [1,2,3,4,5]. However, it is not fully understood what underlying factors contribute to yellow skin appearance.

Bilirubin is a metabolite of senescent red blood cells largely produced in the spleen, bone marrow, and/or liver. Bilirubin is transported in the blood stream, mostly in a form tightly bound to albumin. Free or unbound bilirubin is reported to have the ability to diffuse out of blood vessels and into tissues, where it can act beneficially as an antioxidant [6]. However, bilirubin can also be cytotoxic to cells. For instance, excessive bilirubin accumulation induces neurotoxicity in the jaundice condition frequently occurring in newborn babies, manifesting with signature yellow skin color [7]. Bilirubin formation involves the action of heme oxygenases (HMOXs). Two isoforms of heme oxygenase have been characterized: an inducible form, heme oxygenase-1 (*HMOX1*), and a constitutively expressed form, heme oxygenase-2 *(HMOX2*) [8,9,10,11]. HMOXs catalyze the first rate-limiting step to oxidize and degrade heme into carbon monoxide, ferrous iron, and biliverdin in an equal stoichiometric ratio [12,13]. Biliverdin is subsequently degraded to produce bilirubin by biliverdin reductase. HMOXs are also reported to be induced by a variety of stress factors, such as hypoxia, hyperoxia, proinflammatory cytokines, nitric oxide, heavy metals, ultraviolet (UV) ray radiation, heat shock, shear stress, and hydrogen peroxide (H_2_O_2_) [14,15].

In 1950, London and Gray estimated that 10–20% of bilirubin excreted by humans originates extrahepatically [16,17]. It has been speculated that free heme and porphyrin that are not used for hemoglobin synthesis may be functional resources for bilirubin synthesis outside the liver [7]. Corroborating these initial speculations, recent research has shown that heme synthesis and subsequent bilirubin production can be detected in other cell types of nonhematopoietic origin [18]. In parallel, inhibitors of heme biosynthesis downregulate the bilirubin level [18]. In addition, Numata et al. reported that inflammatory cytokines such as IL-1α, IL-17A, and TNF-α significantly increased *HMOX1* mRNA expression in keratinocytes in association with bilirubin accumulation in the stratum corneum of human epidermis [19]. These studies suggest the possibility that epidermal keratinocytes may be capable of de novo bilirubin synthesis via *HMOX1* induction under stressed conditions.

Here, we hypothesize that human skin keratinocytes house the machinery to perform de novo bilirubin synthesis, especially under oxidative stress, and the synthesized bilirubin may contribute to yellow skin appearance. We also highlight the potential usefulness of the combination of sucrose laurate and dilaurate (SDL) as a novel cosmetic ingredient in managing bilirubin-mediated yellowness.

## 2. Results

### 2.1. Bilirubin-Synthesis-Related Genes Are Expressed in Human Keratinocytes and Upregulated by Oxidative Stress

**Figure 1:**
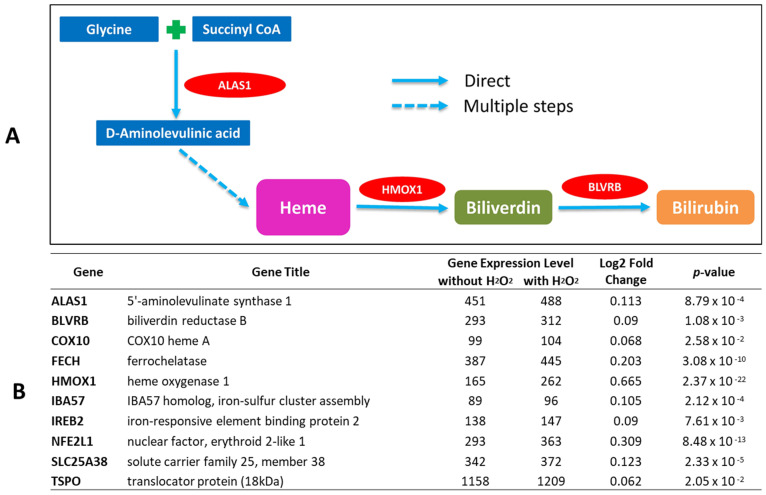
Transcriptome expression of key genes involved in heme and bilirubin synthesis detected from tKC cell culture with and without hydrogen peroxide treatment. (**A**) Simplified diagram of heme biosynthesis and bilirubin production (**B**) List of key genes with transcriptome expression level, log2 fold changes, and *p*-values of hydrogen peroxide treatment group vs. no treatment control group. Genes encoding key enzymes (*ALAS1*, *HMOX1*, and *BLVRB)* were all significantly upregulated by hydrogen peroxide treatment (250 µM, 6 h). *n* = 6/group.

To determine the expression of bilirubin-synthesis-related genes and the impact of oxidative stress, we conducted a transcriptome analysis study using human telomerase reverse-transcriptase-modified immortalized keratinocytes (tKCs) [20]. A simplified diagram of the bilirubin production pathway with three major rate-limiting enzymes encoding genes (*ALAS1, HMOX1*, and *BLVRB*) is shown in Figure 1A. In the tKC keratinocyte monolayer culture, we detected gene expression of various transcripts related to the heme catabolic pathway such as δ-aminolevulinic acid dehydratase (*ALAS*), uroporphyrinogen III synthase (*UROS*), uroporphyrinogen III decarboxylase (*UROD*), oxygen-dependent protoporphyrinogen IX oxidase (*PPO*), and ferrochelatase *(FECH*), as well as de novo bilirubin synthetic pathway gene expression, including biliverdin reductase (*BLVRA*, *BLVRB*), *HMOX1*, and *HMOX2* (Figure 1B). Upon stimulation with H_2_O_2_, the expression of all key genes, including *ALAS*, *HMOX1*, and *BLVRB*, was significantly upregulated (Figure 1B). These results suggested that keratinocytes are equipped with an intracellular mechanism to synthesize bilirubin de novo.

### 2.2. Evidence for De Novo Synthesis of Bilirubin via Heme Synthesis in Human Keratinocytes

**Figure 2:**
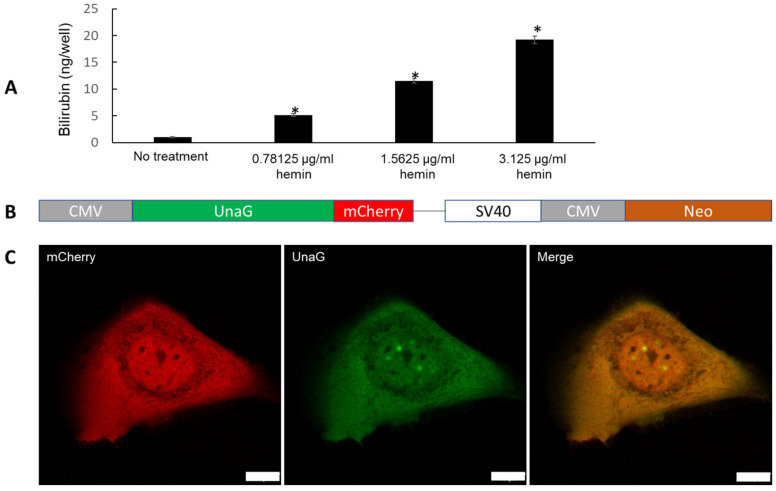
(**A**) Dose–response effect of hemin on de novo bilirubin production in keratinocytes for 48 h. Bar indicates mean ± SEM. *; *p* < 0.05 vs. no treatment group. (**B**) UnaG-mCherry bilirubin sensor. Observation of mCherry allows identification of transfected and expressing cells in absence of bilirubin-induced UnaG fluorescence and enables signal normalization to correct for cell-to-cell expression variation. (**C**) Human keratinocytes expressing sensors grown and maintained in bilirubin-free EpiLife media. mCherry (red) confirms sensor expression. UnaG fluorescence (green) indicates intracellular de novo synthesis of bilirubin in keratinocytes. Scale = 10 µm.

Although the culture media did not contain any bilirubin, we detected marginal levels of bilirubin in the keratinocyte monolayer culture (Figure 2A). Notably, the bilirubin concentration increased in a dose-dependent fashion with the addition of hemin, a natural substrate of *HMOX1* and *HMOX2* (Figure 2A). These results strongly suggested the de novo synthesis of bilirubin in human keratinocytes. The dose-dependent upregulation of bilirubin by hemin further supports the notion that hemin is a natural substrate for HMOXs [21].

Airyscan confocal microscopy was then used for visualizing and detecting bilirubin in keratinocytes transiently transfected with the UnaG-mCherry bilirubin-binding probe (Figure 2B). Although these keratinocytes were maintained in bilirubin-free medium, the transfected mCherry-positive keratinocytes coexpressed UnaG-binding bilirubin (green fluorescence) (Figure 2C). These results further underscored the notion that bilirubin was indeed synthesized by human keratinocytes de novo.

**Figure 3:**
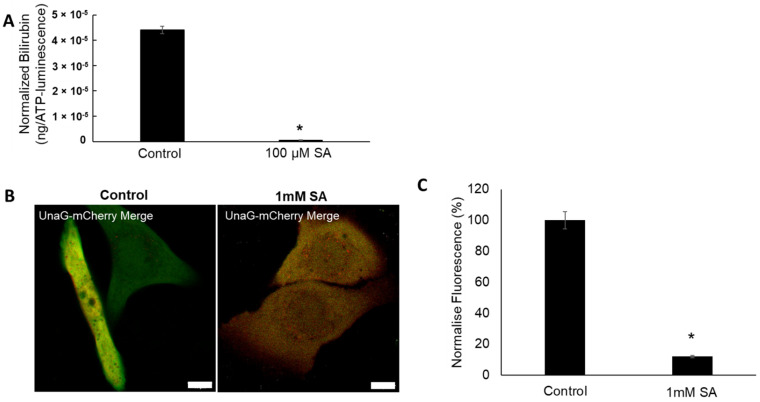
(**A**) De novo bilirubin synthesis in keratinocytes with or without succinyl acetone (SA, an inhibitor of ALA dehydratase) for 48 h. Bar indicates mean ± SEM. *n* = 4/group. *; *p* < 0.05 vs. untreated control group. (**B**) Representative images of untreated control keratinocytes (left, endogenous bilirubin) and keratinocytes treated with SA for 48 h (right). Scale = 10 µm. (**C**) Quantification of UnaG green fluorescence (bilirubin) by image analysis in nontreated control and 1 mM SA-treated keratinocytes for 48 h. SA treatment inhibited intracellular production of bilirubin. Bar indicates mean ± SEM. *; *p* = 0.001 versus control.

We next assessed the role of the heme synthesis pathway in de novo bilirubin production in keratinocytes using succinyl acetone (SA), an inhibitor of ALAS, which is a rate-limiting heme biosynthetic enzyme [15,22] (Figure 1A). As expected, the synthesized bilirubin level was significantly inhibited by SA treatment (Figure 3A).

The effect of SA on intracellular bilirubin expression was further tested with the UnaG-mCherry sensor assay in living keratinocytes. SA inhibited the intracellular expression of bilirubin (Figure 3B,C). These data indicated that ALAS is likely to be one of the major regulatory enzymes for bilirubin production in human keratinocytes.

### 2.3. Enhanced Bilirubin Production by H_2_O_2_ Treatment in Keratinocytes

**Figure 4:**
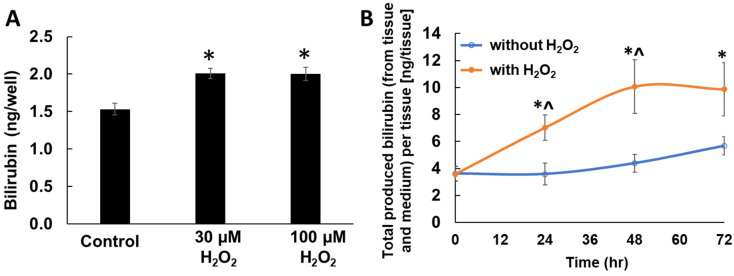
(**A**) Impact of hydrogen peroxide (H_2_O_2_) treatment (48 h) on de novo bilirubin synthesis in keratinocytes. Bar indicates mean ± SEM. *n* = 4/group. *; *p* < 0.05 vs. untreated control. (**B**) Time-course detection of bilirubin in EpiDerm™ 3D human epidermal equivalence cultures (sum of tissue and medium) with and without 30 µM hydrogen peroxide (H_2_O_2_) in medium for 72 h. Bilirubin was quantified using HPLC-MS method. Data are mean ± SEM. *n* = 4/group. *; *p* < 0.05 vs. baseline, ^; *p* < 0.05 vs. without H_2_O_2_ group.

To investigate the impact of oxidative stress on keratinocyte-derived bilirubin production, the monolayered keratinocytes were treated with H_2_O_2_ for 48 h, followed by bilirubin quantification using HPLC-MS/MS. The noncytotoxic levels of H_2_O_2_ significantly upregulated bilirubin production (Figure 4A), suggesting that oxidative stress could upregulate the de novo synthesis of bilirubin in keratinocytes.

We next confirmed bilirubin production and its augmentation by H_2_O_2_ using EpiDerm™ (MatTek Co., Ashland, MA, USA), a commercially available, reconstituted, three-dimensional (3D) human skin-equivalent model. No bilirubin was detected from fresh medium. Bilirubin was readily detected in untreated control skin-equivalents (Figure 4B). Upon H_2_O_2_ treatment, bilirubin levels were significantly increased at both 24 h and 48 h compared with untreated control samples, while significance was lost at 72 h (Figure 4B). This increasing trend of bilirubin level is consistent with the results in the aforementioned monolayer keratinocyte culture.

We also calculated the net amounts of bilirubin produced in the 3D human skin-equivalent model (Table 1). The untreated control samples showed bilirubin levels ranging from 1.4 to 2.3 µg/mL, with an increasing pattern at a later time point, while H_2_O_2_-treated groups showed significantly higher bilirubin levels, ranging from 2.8 to 4.0 µg/mL, than controls (Table 1). These results suggested that bilirubin was generated even in the well-differentiated 3D skin-equivalents, and its production was significantly enhanced by oxidative stress.

### 2.4. Bilirubin Has Natural Affinity toward Epidermis of Human Skin

**Figure 5:**
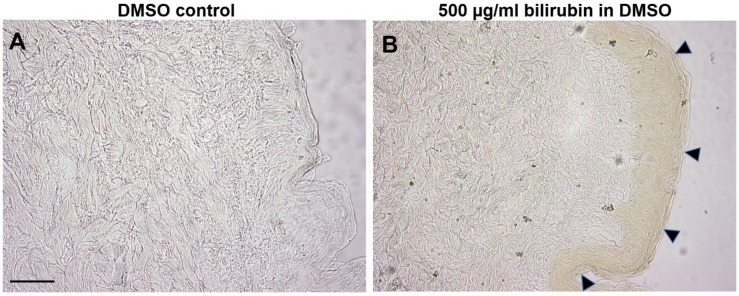
Bright-field microscopy of human abdominal skin section (8 µm) incubated with either (**A**) 100 µL of DMSO or (**B**) 500 µg/mL bilirubin dissolved in DMSO for 1 h in dark. Arrowheads indicate visible bilirubin (yellow color) accumulation in epidermal layer of skin section. Scale bar = 100 µm.

Bilirubin is a lipid acid and is naturally lipophilic. To determine whether bilirubin has a higher affinity for the epidermal layer and stratum corneum due to its high lipid content, frozen human skin sections were incubated with 500 µg/mL unconjugated bilirubin. As shown in Figure 5, bilirubin applied to thin sections of human skin showed preferential accumulation to the epidermis and the stratum corneum, resulting in visibly yellow staining. Considering its lower accumulation in the dermal compartment, bilirubin may be more capable of binding to the epidermis than the dermis.

### 2.5. Assessment of Yellowness (b* Value) in Human Epidermal Explant Model Treated with Bioavailable Concentrations of Bilirubin

**Figure 6:**
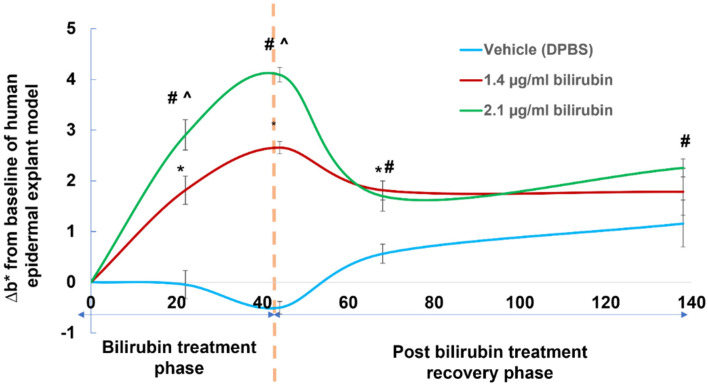
Human epidermal skin explants were cultured with vehicle, 1.4 µg/mL bilirubin or 2.1 µg/mL of bilirubin for 44 h (treatment phase). At 44 h, the bilirubin treatments were replaced with DPBS vehicle without bilirubin and epidermal skin samples were equilibrated till 138 h (recovery phase). Data indicate mean ± SEM. *n* = 6/group. *; *p* < 0.05 of 1.4 µg/mL bilirubin leg vs. vehicle-treated control sample. #; *p* < 0.05 of 2.1 µg/mL bilirubin leg vs. vehicle-treated control sample. ^; *p* < 0.05 for 2.1 µg/mL group vs. 1.4 µg/mL group.

To assess the impact of bilirubin in the epidermis on skin appearance, epidermal human skin explants were incubated with bioavailable levels of bilirubin (1.4 or 2.1 µg/mL) for 44 h in a vehicle of DPBS buffer. Afterward, skin samples were equilibrated in DPBS buffer without bilirubin for 138 h. Changes in yellowness scores from baseline (Δb* values) were measured at 0, 22, 44, 68, and 138 h after the initiation of culture (Figure 6). Bilirubin treatment enhanced ∆b* values in a time- and dose-dependent manner compared with the control vehicle during the bilirubin treatment phase (Figure 6). After 44 h (recovery phase without bilirubin treatment), the ∆b* values of bilirubin-treated legs (both 1.4 and 2.1 µg/mL) gradually decreased to baseline levels between 68 and 138 h after the initiation of culture. These results further underpinned the notion that bilirubin effectively, but transiently, binds human skin explants and increases their yellowness.

### 2.6. Transcriptomic Profiling Analysis of Keratinocytes by Bilirubin Treatment

**Figure 7:**
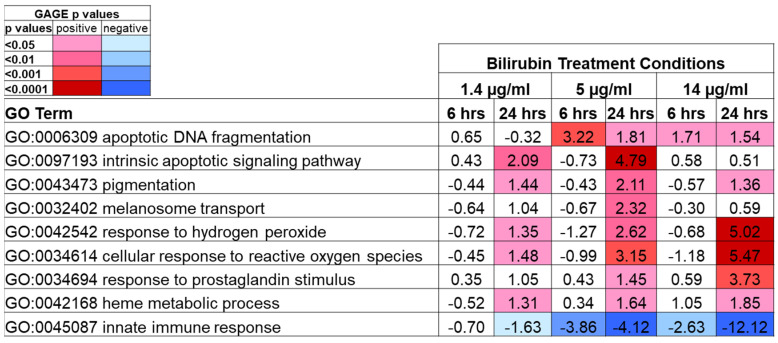
Key modulated biological pathways based on Gene Set Enrichment Analysis treated with different concentrations of bilirubin on human neonatal keratinocytes. Data are shown as log10 of GAGE *p*-values (negative represents inhibition, positive represents activation). *n* = 8/group. Color code indicates *p*-value range.

Bilirubin is well known to act as a chemical antioxidant and is thought to offer cytoprotection [23]. On the other hand, high levels of bilirubin are known to induce neurotoxicity, as represented in jaundice [7]. To elucidate the functional properties of bilirubin, we treated keratinocytes with 1.4, 5, or 14 µg/mL of bilirubin and performed a transcriptome profiling assay. We found significant upregulation of gene sets related to “response to oxidative stress”, “prostaglandin stimulus”, and “heme metabolic process” pathways in a dose-dependent manner, mostly at 24 h time points across all bilirubin concentrations (Figure 7). In addition, we observed significant gene upregulation in the pathways of “apoptosis” and “skin pigmentation”, with significant downregulation of genes in “innate immune response” even at the lowest bilirubin concentration (1.4 µg/mL). These results suggested that bilirubin has broader biological potential beyond oxidative stress response.

### 2.7. SDL Diminishes Bilirubin Levels in Keratinocytes

**Figure 8:**
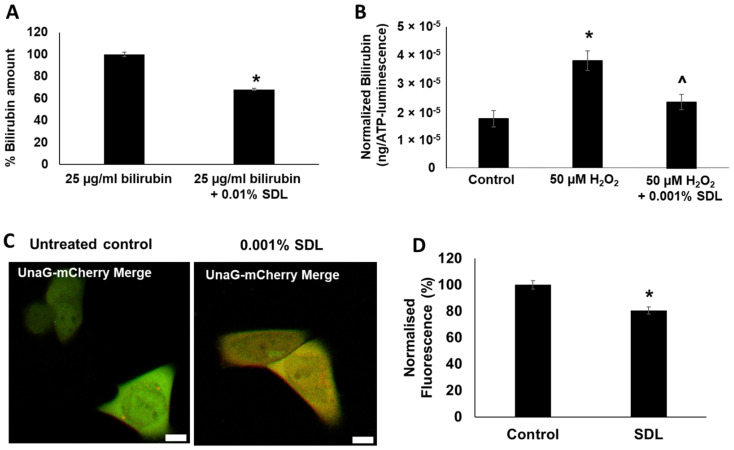
(**A**) Reduction of bilirubin concentration by incubation with 0.01% SDL for 20 h. Bar indicates mean ± SEM. *n* = 3/group. *; *p* < 0.05 vs. control. (**B**) Inhibitory effect of 0.001% SDL on H_2_O_2_-induced bilirubin production in keratinocytes at 48 h. Bilirubin concentrations were quantified using HPLC-MS method and were normalized to ATP level (an indicator of cell viability). Bar indicates mean ± SEM. *n* = 4/group. *; *p* < 0.05 vs. control. ^; *p* < 0.05 vs. 50 µM H_2_O_2_-treated group. (**C**) Visualization of bilirubin production by using UnaG-mCherry bilirubin sensor in keratinocytes in absence or presence of 0.001% SDL for 48 h. Scale = 10 µm. (**D**) Calculated green fluorescence intensity (bilirubin) normalized to red fluorescence intensity (mCherry). Bar indicates mean ± SEM. *n* = 13/group. *; *p* < 0.01.

In order to mitigate skin yellowness induced by bilirubin, we screened over 100 skin care materials and identified SDL as the most potent compound to reduce bilirubin. In a chemical in vitro assay, the addition of 0.01% SDL significantly reduced the bilirubin level within 20 h (Figure 8A). Furthermore, in the keratinocyte monolayer culture model, SDL significantly reduced H_2_O_2_-induced bilirubin production (Figure 8B). The effect of SDL treatment on intracellular bilirubin was also visualized and quantified in human keratinocyte cell cultures using the UnaG-mCherry bilirubin sensor (Figure 8C,D). SDL treatment significantly reduced the intracellular levels of bilirubin (Figure 8C,D).

**Figure 9:**
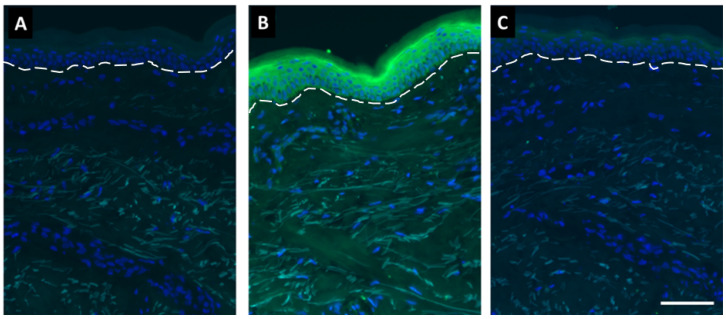
Fluorescent images of frozen human skin sections. Green color in epidermis is bilirubin; blue color indicates DAPI staining. Green signals observed in dermis in nontreated control are due to autofluorescent property of elastin. White dotted line indicates dermal–epidermal junction: (**A**) nontreated control; (**B**) 50 µg/mL bilirubin; (**C**) 50 µg/mL bilirubin + 0.01% SDL. Scale bar = 100 µm.

Finally, we assessed the effects of SDL on skin-bound bilirubin. Frozen human skin sections were incubated with bilirubin with or without SDL. The baseline fluorescence intensity of untreated skin sections was marginal (Figure 9A). The clear green fluorescence of skin-bound bilirubin was visualized, especially in the epidermal compartment (Figure 9B). Notably, SDL markedly ameliorated bilirubin-induced epidermal fluorescence (Figure 9C). These results indicated that SDL is a useful technology to reduce skin-bound bilirubin content.

## 3. Discussion

Positioned at the interface of the body and the external environment, the skin is a protective barrier that is constantly under attack by various stresses from both the external environment and the internal biological system [24]. The skin is routinely exposed to environmental damage such as UV light and pollution, resulting in reactive oxygen species generation. The skin is also exposed to oxidative conditions derived from internal biological responses due to physical or mental stresses [25,26]. To maintain homeostasis, the skin is equipped with a network of redox antioxidative systems, including antioxidative enzymes such as catalase and superoxide dismutase, as well as chemical antioxidants such as glutathione [24,27].

It has been reported that bilirubin has cytoprotective functions by acting as an effective antioxidant against lipid peroxidation [23]. Our data strongly indicate that epidermal keratinocytes not only house all elements needed for de novo synthesis of heme and bilirubin, but the mechanism does indeed produce bilirubin via the heme synthesis pathway (Figure 1, Figure 2 and Figure 3). We also demonstrated that skin bilirubin synthesis is upregulated by oxidative stress (Figure 4). However, further studies are warranted to elucidate whether the increased production of bilirubin is attributable to increased enzymatic activity or the increased protein level of enzymes.

The presented evidence is well corroborated with the documented response of *HMOX1* induction and detection of bilirubin in the stratum corneum [19]. In the present study, the average bilirubin concentration calculated from the 3D human skin-equivalent model was 1.4 µg/mL (baseline level), which was significantly elevated to 4.01 µg/mL on average by oxidative stress (Table 1). The levels are comparable to the average serum levels of circulating bilirubin (5.2 µg/mL) in healthy women [28]. This evidence indicates that the human skin, specifically epidermal keratinocytes, is an active and functional source of extrahepatic bilirubin production. In addition, this study demonstrated the preferential accumulation of bilirubin in the epidermal compared to the dermal compartment. We do not know if this phenomenon is specific to the biological nature of bilirubin. Further studies are necessary to clarify this mechanism. However, there is a possibility that bilirubin produced from epidermal keratinocytes may remain in the epidermis. This is intriguing due to the fact that the epidermis is not directly connected to the blood circulatory system and thus is less exposed to circulating bilirubin produced elsewhere in the body.

However, bilirubin has an aesthetically negative impact with its intrinsic yellow color. Yellow skin color is associated with poor health. Cosmetically, it is associated with negative characteristics such as sallow or dull skin appearance. In parallel, we found that bilirubin had a high tendency to bind to human skin, especially to the epidermis (Figure 5). The yellowish hue of skin explants was increased by incubation with bioavailable bilirubin levels, which were detected in H_2_O_2_-treated skin-equivalents. The increased yellowness gradually subsided to the baseline level after eliminating bilirubin in the treatments (Figure 6). The fact that bilirubin had high selectivity toward the epidermal compartment may strengthen the role of bilirubin in yellowish skin tone.

Oxidative stress can be caused by many factors, including UV radiation, exposure to environmental pollutants such as cigarette smoke, or sleep disruption. Much attention has been paid to the impact of UVB on the skin, as well as the oxidative stress of cigarette smoke, leading to the enhancement of yellowness around the mouth and hands of smokers. Our data suggest a possibility that oxidative stress from cigarette smoke may induce bilirubin production that partially contributes to yellowness. Sleep deprivation/disruption is a very common concern as a byproduct of modern life demands. Sleep disturbance is reported to induce oxidative stress [29], increase the serum levels of bilirubin [30] and even cause liver disorder [31]. Furthermore, there are various papers suggesting bilirubin induction by inflammatory cytokines [1,2,3,4,5,32]. In addition, our transcriptomic analysis showed that keratinocytes treated with a high level of bilirubin manifest oxidative stress responses (Figure 7), which is consistent with the literature, showing bilirubin induces oxidative stress and causes DNA damage at high concentrations [33]. The combination of published work and our data strongly suggests that increased bilirubin formation can be an indicator of stressed skin, which correlates with the historical view that yellow skin equals unhealthy skin.

SDL has been widely used as a safe emulsifier or stabilizer in cosmetic, food, and pharmaceutical industries for many years. The present study demonstrated that SDL significantly reduced bilirubin levels and diminished the skin-bound bilirubin concentration (Figure 8 and Figure 9). Although further research is required to elucidate the detailed mechanism of action, preliminary transcriptome analysis in human keratinocytes indicated SDL upregulates UDP-glucuronosyltransferase 1A1 (UGT1A1), which is the key enzyme catalyzing bilirubin conjugation to sugars and results in increased water solubility of bilirubin and enhanced elimination via bile and urine [34].

In conclusion, we showed direct evidence that epidermal keratinocytes are capable of producing bilirubin and that keratinocyte-derived bilirubin synthesis is augmented by oxidative stress. In addition, the predominant binding of bilirubin to the epidermis increases yellowish discoloration. As the yellow component of skin tone is attributable to the sallow and/or dull appearance of facial skin, bilirubin production from epidermal keratinocytes may directly cause facial skin discoloration. We also demonstrated that SDL is effective in reducing bilirubin in various in vitro models. SDL could be a potential measure to prevent bilirubin-mediated unhealthy yellowish/dull skin appearance after oxidative stress.

## 4. Materials and Methods

### 4.1. Chemicals, Reagents, and Cell Lines

Culture medium and supplements were all purchased from Thermo Fisher Scientific (Waltham, MA, USA), including EpiLife calcium-free phenol red-free medium (Cat. No.: MEPI500CA), gentamicin/amphotericin B (500X; Cat. No.: 50-0640), calcium chloride (Cat. No.: 50-9703), HKGS (100X; Cat. No.: S-001-5), trypsin/EDTA solution (TE; Cat. No.: R001100), trypsin neutralizer solution (TN; Cat. No.: R002100), penicillin/streptomycin (10,000 U/mL, 100X; Cat. No.: 15140122), and DPBS (Cat. No.: 14190250). The keratinocyte cell line tKC was a kind gift from Dr. Shay (University of Texas Southwestern, Dallas, TX) [20]. ViaStain AO/PI staining solution was purchased from Nexcelom Bioscience (Cat. No.: CS2-0106-5 mL, Lawrence, MA, USA) and AccuGene 1x PBS from Lonza (Cat. No.: 51225, Alpharetta, GA, USA). Other chemicals used for all studies, including DMSO (Cat. No.: D8418-100 mL), H_2_O_2_ (30% stock in water; Cat. No.: 216763-100mL), hemin (Cat. No.: H9039-1G), and 4,6-dioxoheptanoic acid (succinyl acetone (SA)) (Cat. No.: D1415-500 mg) were purchased from Sigma (St. Louis, MO, USA). Bilirubin (Cat. No.: 17161) and bilirubin conjugate (sodium salt; Cat. No.: 17170), were purchased from Cayman Chemicals (Ann Arbor, MI, USA). CellTiter-Glo^®^ luminescent cell viability assay kit (Cat. No.: G7571) was purchased from Promega (Madison, WI, USA). SDL (combination of sucrose laurate and sucrose dilaurate at approximately 6:4 ratio) was supplied from BASF (Ludwigshafen, Germany).

### 4.2. tKC Culture, Treatment, and Viability Assessment

tKCs were plated in 24-well tissue culture plates using MEPI500CA media supplemented with HKGS and gentamicin/amphotericin B at 200,000 cells/well. The cells were incubated at 37 °C under 5% CO_2_ and 95% humidity for 24 h before treatment. All treatments were made in MEPI500CA medium that contained 1 mM added calcium chloride and 0.1% DMSO. After treatment, the cultures were maintained at 37 °C under 5% CO_2_ and 95% humidity for 48 h before harvesting. When harvesting, the culture supernatant was transferred onto a fresh 24-well plate and stored on dry ice immediately and shielded from light. Cell layers were also stored on dry ice immediately and shielded from light. Both the cell layer plate and medium plate were subsequently frozen at −80 °C until quantifying for bilirubin.

For viability assessment, the CellTiter-Glo^®^ luminescent cell viability assay was used according to the manufacturer’s instructions. Briefly, CellTiter-Glo^®^ reagent was mixed with an equal volume of cell culture medium and vortexed to create a homogeneous Cell GLO viability working solution. After removing cell culture media, tKCs were treated with 300 µL of the Cell GLO viability working solution. The plate was covered with aluminum foil and agitated on an orbital shaker at 150 rpm for 10 min. The plate was then read with a SpectroMax microplate reader (Molecular Device, San Jose, CA, USA) for chemiluminescence intensity.

### 4.3. Oxidative Stress Challenge to 3D Human Skin-Equivalent Model

EpiDerm™ 3D human skin-equivalent cultures were purchased from MatTek corporation (MatTek, Ashland, MA, USA) and cultured according to the manufacturer’s recommendations. EpiDerm™ cultures contain highly differentiated human-derived epidermal keratinocytes. The EpiDerm™ 3D cultures were immediately incubated with phenol red-free medium (Part No.: EPI-100-ASY-PRF) at 37 °C with 95% humidity and 5% CO_2_ for 20 h to equilibrate. A baseline group of 4 tissues and corresponding culture supernatant were harvested on dry ice and shielded from light before transferring to −80 °C for storage. The rest of the tissues were subject to either medium treatment as control groups or 30 µM H_2_O_2_ treatment as oxidatively stressed groups (*n* = 4 per group). All tissues were incubated at 37 °C with 95% humidity and 5% CO_2_ until the designated harvesting time points: 24 h, 48 h, and 72 h.

### 4.4. UnaG Bilirubin Quantification Method for Cultured tKC

UnaG is a Japanese Eel muscle protein. It binds bilirubin, forming a complex that emits green fluorescence, making it a good bilirubin sensor [35]. The UnaG-His-FLAG recombinant protein used in this study was obtained from Riken Institute (Tokyo, Japan) [36]. The bilirubin detection method by UnaG was reported previously [37]. Briefly, bilirubin was extracted from tKC cells cultured on a 24-well plate with a combination of 110 µL of 0.1% triethanolamine sourced from Univar USA Inc (Bedford Park, IL, USA) and 110 µL of 1 µM UnaG stock made in PBS. To prevent bilirubin photodegradation, the plate was covered with aluminum foil. The plate was shaken by a VWR Signature™ Digital Multi-Tube Vortexer (VWR, Batavia, IL, USA) at 2000 rpm for 20 min to extract bilirubin. A bilirubin standard curve was obtained covering the range of 9.13 to 292.33 ng/mL. UnaG concentration was 0.5 µM for all standards and samples. Fluorescence intensity proportional was read using a SpectroMax with fluorescence filters for excitation and emission wavelengths of 497 and 527 nm, respectively. Bilirubin concentrations were calculated by linear regression against the bilirubin standard curve.

### 4.5. Bilirubin Quantification to Assess SDL Chemical Impact on Bilirubin Level

The effect of SDL on bilirubin level was assessed with 20 h incubation in a cell-free system. Three replicates of each test sample were prepared on a 96-well plate (e.g., a FALCON brand 96-well tissue culture plate or equivalent) at a total volume of 250 μL/well. Positive control wells contained 25 µg/mL bilirubin in PBS buffer (AccuGENE, Cat. No.: 51225), while negative/vehicle control wells contained DMSO and PBS buffer. SDL was dissolved in DMSO. Each test sample well contained 25 µg/mL bilirubin with SDL added to 0.01% in PBS buffer. The plates were covered with aluminum foil and placed on top of a microplate shaker (VWR, Cat. No.: 12620-938), followed by incubation at room temperature for 20 h with constant shaking at 150 rpm. Bilirubin was then quantified after incubation using a commercially available bilirubin quantification kit (Cat. No.: MET-5010, Cell Biolab, San Diego, CA, USA). Briefly, quantitation is based on the Jendrassik–Grof method using diazotized sulfanilic acid to react with bilirubin to form azobilirubin, detected at an OD of 540 nm. A standard bilirubin curve is generated. Bilirubin concentrations of all testing legs are calculated using linear regression against the bilirubin standard curve. Bilirubin reduction activity was determined by comparing bilirubin levels of treatments vs. the bilirubin-positive control leg.

### 4.6. Human Epidermal Explant Model Treatment with Bilirubin

Twelve-millimeter punch biopsies of human abdominal explant (46 years, female, skin type III) were procured from cosmetic procedures with an IRB-approved protocol (Schulman Associates Institutional Review Board, Cincinnati, OH, USA). After fat removal, the skin was cut into 1.25 cm^2^ squares, placed in 1 M NaCl plus 10× penicillin/streptomycin (Invitrogen), and incubated overnight at 37 °C. The following day, the epidermis was carefully peeled off with forceps and stored in phosphate-buffered saline (PBS) plus 2× penicillin/streptomycin and stored at 4 °C before use (Bachelor et al., 2014). To assess bilirubin’s impact on skin tone appearance, the epidermal explants were cultured in individual inserts that sat on top of 6-well culture plates. Tissues were simultaneously treated both topically (50 µL) and in the medium 6-well plates (2.3 mL per well) with one of the 3 treatments: control vehicle (DPBS, Thermo Fisher), 1.4 µg/mL conjugated bilirubin solution, and 2.1 µg/mL conjugated bilirubin (sodium salt, Cayman Chemicals) solution, respectively. Epidermal explant tissues were imaged with a Spectroshade imaging device (SpectroShade USA, Oxnard, CA, USA) at designated time points and b* values (yellowness score) were quantified from the images using built-in software.

### 4.7. Bilirubin Quantification in Biological Samples Using LC-MS/MS

Biological samples were extracted with 0.1% triethylamine (TEA) to recover synthesized bilirubin. Briefly, 0.1% TEA was added into 3D human skin-equivalent tissue samples (120 µL per tissue) or monolayer cells in the well plate (110 µL per well of a 24-well cell culture plate), transferred to low-binding Eppendorf tubes, followed by vortexing for 10 min at room temperature. Samples were then centrifuged at 16,000× *g* using a benchtop centrifuge for 10 min with the supernatants used for analysis. The standards and the samples were analyzed using gradient high-performance liquid chromatography with tandem mass spectrometry (HPLC-MS/MS). Bilirubin can be separated by a reverse-phase column (Atlantis T3 Column, 100Å, 3 µm, 2.1 mm × 50 mm; Waters, Milford, MA, USA). Bilirubin and the corresponding stable isotope-labeled internal standard (ISTD) were monitored by electrospray ionization (ESI) in positive mode using the selected reaction monitoring schemes below.

- Multiple reaction monitoring (MRM) transitions for analytes (bilirubin: 585.2 → 299.2);
- Their corresponding stable isotope-labeled internal standards (d4-bilirubin: 589.2 → 301.2).

The Used ISTD was d4-bilirubin (Toronto Research Chemicals, North York, ON, Canada). A standard curve was constructed by plotting the signal, defined here as the peak area ratio (peak area analyte/peak area ISTD), for each standard versus the concentration of each analyte for the corresponding standard. The concentration of bilirubin in the calibration standards and biological samples were then calculated using the generated regression equation.

### 4.8. Estimation of Bilirubin Concentration Produced in EpiDerm™ 3D Human Skin-Equivalent Model

The diameter of each 3D human skin-equivalent model tissue is 8 mm, and the thickness is about 50 µm. By using such dimensions, an individual 3D human skin tissue volume was calculated as: π × (0.4 cm)^2^ × 0.005 cm = 0.002513 cm^3^ = 0.002513 mL. Upon normalizing the sum of bilirubin produced by each tissue, i.e., factor in bilirubin quantified from both tissue itself and corresponding culture supernatant/medium, the mean bilirubin concentration per milliliter tissue volume of each group was calculated.

### 4.9. Sample Preparation and Data Analysis for Microarray Studies

To investigate the impact of ROS stress, tKC cells were plated out at a density of 250,000 cells/well into 12-well plates. After growing for 24 h at 37 °C in a CO_2_ incubator, the tKCs were treated with a media control vehicle and hydrogen peroxide at 250 µM for 1 h, respectively, before harvesting 6 h later for microarray analysis. To investigate the impact of bilirubin treatment on human primary keratinocytes, a control vehicle (10% water) and conjugated bilirubin at 1.4 μg/mL, 5 μg/mL, or 14 μg/mL, were added respectively, before harvesting for microarray analysis at 6 h and 24 h. Samples were collected in RNAlater^®^ buffer, flash-frozen, and stored at −80 °C prior to RNA extraction. RNA was extracted and purified using the RNeasy kit (QIAGEN, Germantown, MD, USA). Purified RNA was converted to biotin-labeled complementary RNA copies using the HT 3′ IVT Plus kit (Affymetrix, Santa Clara, CA, USA), per the manufacturer’s protocol. Biotinylated cRNA was fragmented by limited alkaline hydrolysis and then hybridized overnight to Affymetrix GeneTitan U219 array plates using the Affymetrix GeneTitan instrument and protocol. Probe set expression values were calculated by quartile normalization and PLIER summarization algorithms. Differentially expressed genes were analyzed using the ANOVA model implemented in the R limma package [38]. Gene Set Enrichment Analysis (GSEA) was performed using GAGE (Generally Applicable Gene set Enrichment for pathway analysis) model [39] against Gene Ontology datasets.

### 4.10. Visualization of Bilirubin Content within Cells Using UnaG Fluorescence

HaCaT keratinocytes were cultured in EpiLife medium (Thermo Fisher). At 72 h prior to imaging, cells were seeded in 35 mm quad No1.5 polymer coverslip-bottomed dishes (Ibidi) and grown to 70% confluency at the time of transfection. At 48 h prior to imaging, the cells were transfected with the CMV-UnaG-mCherry construct (Figure 2B) using Lipofectamine 3000 (Thermo Fisher). Transfection materials were added to Optimem Reduced Serum medium (Thermo Fisher) and added to cells for 4 h before the medium was removed and replaced with fresh media. Treatments were added to the cells at this stage for 48 h. Immediately prior to imaging, the media was removed, and Fluorobrite medium (Gibco) was added. All live-cell imaging was performed on a Zeiss LSM 880 confocal microscope with the Airyscan detector with either a 20X 0.8 NA Plan-Apochromat air lens or a 63X 1.4NA Plan-Apochromat oil immersion lens. The imaging environment was maintained at 37 °C and 5% CO_2_. UnaG was excited using a 488 nm laser and emission captured by the Airyscan detector with a 495–550 nm bandpass filter. mCherry was excited using a 594 nm laser and emission captured by the Airyscan detector with a 645 nm long pass filter. Mean fluorescent intensity of cells was measured using ImageJ. Individual cells were isolated by ROI and the mean fluorescence intensity of each channel measured. UnaG intensity values are expressed as a proportion of the mCherry signal to normalize and control for variation in probe expression.

### 4.11. Visualization of SDL Effect on Bilirubin Reduction in Human Skin Sections

A 10 µm amount of fresh frozen human skin sections was dried at RT for 30 min, followed by rehydration with PBS. Sections were incubated either with or without (negative control) 50 µg/mL bilirubin 1 h at RT in the dark, followed by washing with PBS. Sections were then treated with either (1) vehicle—0.5% DMSO in PBS or (2) 0.01% SDL in vehicle overnight at RT in the dark, washed in PBS, and DAPI counterstained using NucBlue fixed cell stain Ready Probes reagent (Invitrogen, Carlsbad, CA, USA). For comparison, fluorescent images were captured with a Zeiss Observer.Z1 microscope (Carl Zeiss Microimaging, Jena, Germany) at equal gamma values, pixel ranges, and exposures.

### 4.12. Statistical Analysis

Statistical significance for all in vitro experiments, except for the transcriptome analysis, was determined by Student’s *t*-test unless stated otherwise. Values of *p* < 0.05 were considered statistically significant.

## Figures and Tables

**Table 1:** Time-course changes in total bilirubin (BR) concentration with and without 30 µM hydrogen peroxide treatment in MatTek EpiDerm™ 3D human skin-equivalent model. Tissue volume was estimated as 0.0025 mL/tissue based on assumption of 8 mm in diameter, 50 µm in thickness. Data are mean ± SEM. *n* = 4/group.

Treatment—Time	Total BR Normalized to Estimated Tissue Volume (g/tissue-mL)	*p*-Value	
		vs. Baseline	vs. Corresponding Control Group
Baseline	1.44 ± 0.22	-	-
No H_2_O_2_—24 h	1.43 ± 0.32	0.970	-
No H_2_O_2_—48 h	1.75 ± 0.26	0.403	-
No H_2_O_2_—72 h	2.26 ± 0.27	0.056	-
30 µM H_2_O_2_—24 h	2.80 ± 0.37	***0.020***	***0.031***
30 µM H_2_O_2_—48 h	4.01 ± 0.79	***0.021***	***0.035***
30 µM H_2_O_2_—72 h	3.93 ± 0.78	***0.022***	0.091

## Data Availability

The data presented in this study are available on request from the corresponding author.
